# Which Is More Likely to Achieve Cardiac Synchronization: Left Bundle Branch Pacing or Left Ventricular Septal Pacing?

**DOI:** 10.3389/fcvm.2022.845312

**Published:** 2022-03-28

**Authors:** Kailun Zhu, Dong Chang, Qiang Li

**Affiliations:** ^1^Department of Cardiology, Xiamen Cardiovascular Hospital of Xiamen University, School of Medicine, Xiamen University, Xiamen, China; ^2^School of Medicine, Xiamen University, Xiamen, China

**Keywords:** left bundle branch pacing, left ventricular septal pacing, cardiac resynchronization therapy, right ventricular pacing, physiological pacing

## Introduction

In advanced heart failure patients with low left ventricular ejection fraction and left bundle branch block (LBBB), cardiac resynchronization therapy (CRT) *via* stimulation of both the right ventricle (RV) and the left ventricular lateral wall is a recommended therapeutic strategy (1–3). However, conventional biventricular pacing causes a dyssynchronous cardiac contraction due to non-physiological fusion of paced propagation, with a non-response rate of up to 30% (4, 5). In 2016, Mafi-Rad et al. (6) established the viability of the left ventricular septal pacing (LVSP) *via* a trans-interventricular septal approach in 10 patients with sinus node dysfunction, which shortened QRS duration and preserved acute left ventricular contractility compared to RV pacing. Huang et al. refined LVSP and introduced first left bundle branch pacing (LBBP) in 2017 (7), which could restore physiological left ventricular contractility in a patient with LBBB by pacing left bundle branch (LBB) immediately beyond the conduction blockage with satisfactory pacing parameters. Many studies have demonstrated the feasibility and stability of LBBP in patients with pacemaker indications, and it has been proposed that LBBP is a novel physiological pacing method for delivering CRT for achieving electric resynchronization in patients with LBBB (8–10).

## Brief Pacing Mechanisms of LBBP and LVSP

**Figure 1:**
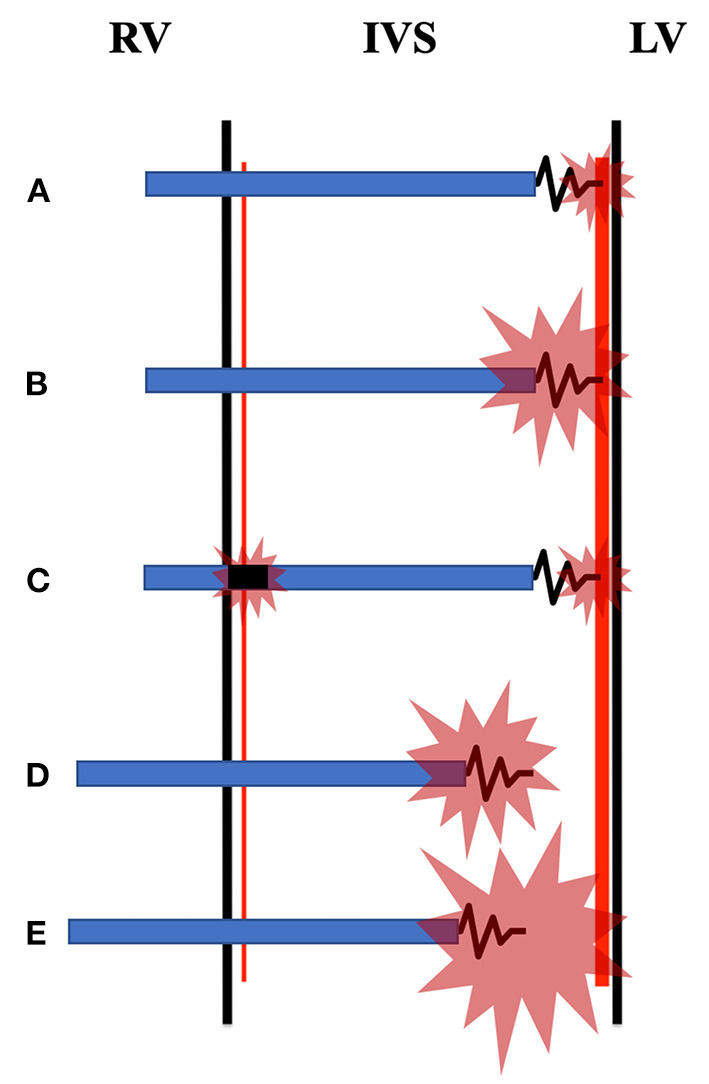
Diagrammatic sketch of left bundle branch pacing (LBBP) and left ventricular septal pacing (LVSP) with different pacing modes. **(A)** Selective LBBP (SLBBP) captures only the left bundle branch (LBB) trunk or its proximal bundle, resulting in physiologically left ventricle (LV) excitation but delayed right ventricle (RV) excitation. Non-selective LBBP (NSLBBP) is divided into two types: **(B)**, which excites the LBB and surrounding myocardium, and **(E)**, which excites the LBB and the left ventricular septal myocardium. **(C)** In bipolar pacing configuration, the cathode tip of LBBP captures the LBB and the anode ring captures the right bundle branch at the same time, probably due to the anodal capture. **(D)** LVSP captures only the left ventricular septal myocardium. RV, right ventricle; IVS, interventricular septum; LV, left ventricle.

Selective LBBP (SLBBP) and non-selective LBBP (NSLBBP) are two subgroups of LBBP. SLBBP, that is, only the LBB trunk or its proximal fascicles is captured (Figure 1A). NSLBBP, that is, concomitant LBB and adjacent myocardium are captured (Figures 1B,E). It is LVSP if just the left ventricular septal myocardium is captured (Figure 1D). Both LVSP and LBBP usually present a paced pseudo right bundle branch block (RBBB) pattern in lead V1 (11), with the percentage of direct evidence that LBBP captured LBB ranging between 60 and 90% (12–14). Therefore, LBBP described in some previous studies was actually LVSP. A method to measure the time from stimulus to left ventricular activation at high and low outputs in lead V5 or V6 (Stim-LVAT) to distinguish LBBP from LVSP with a specificity of 100% has recently been presented (11). If the Stim-LVAT remains shortest and constant (prolonged ≤ 10 ms) as the pacing output decreases, it must be LBBP, because LBBP directly captures the LBB resulting in physiologically LV excitation; otherwise LVSP can be considered, because LVSP excites left ventricular septum first, rather than LBB. SLBBP and NSLBBP can be distinguished by the discrete component and isoelectric interval between the pacing artifact and V wave on intracardiac electrogram with unchanged Stim-LVAT (11).

## Comparison of LBBP and LVSP in Interventricular Synchrony

In the paper published in *Frontiers in Cardiovascular Medicine*, Curila et al. (15) used ultra-high-frequency electrocardiography to compare ventricular depolarization in SLBBP, NSLBBP, and LVSP in 57 bradycardia patients, which were rigorously distinguished by Stim-LVAT. They concluded that LVSP preserved interventricular synchrony and had the same or better local depolarization durations than NSLBBP and SLBBP. Furthermore, they investigated two different types of NSLBBP capture, namely, NSLBBP with LBB and adjacent myocardium captured (Figure 1B), and NSLBBP with LBB and left septal myocardium captured (Figure 1E). NSLBBP with LBB and adjacent myocardium captured, that is, NSLBBP is converted to SLBBP with a shortest and constant Stim-LVAT while decreasing the pacing outputs. NSLBBP with LBB and left septal myocardium captured, that is, NSLBBP is converted to LVSP with prolonged Stim-LVAT while decreasing the pacing outputs. They evaluated the two types of NSLBBP capture and found no statistical difference in Stim-LVAT between the two types, but NSLBBP with LBB and left septal myocardium captured showed greater interventricular synchronization.

Then, which pacing strategy is more physiological, LBBP or LVSP? SLBBP and NSLBBP, unlike LVSP, capture the intrinsic conduction system and rapidly excite LV to maintain left ventricular synchrony at levels comparable to intrinsic left ventricular activation (16). At the same time, activation propagates slowly from left to right in the interventricular septum to excite RV, resulting in interventricular dyssynchrony. LVSP, on the other hand, captures left ventricular septal myocardium, resulting in direct left-to-right septal activation, preserving interventricular dyssynchrony. The terminal R′/r′ wave duration in lead V1, which indicates delayed right ventricular excitation, was significantly longer in LBBP than in LVSP (17), also indicating that LBBP caused more pronounced interventricular dyssynchrony than LVSP. However, this interventricular synchrony of LVSP may not be physiological. Instead of using the same stimulation marker, such as the pacing artifact, Curila et al. calculated interventricular dyssynchrony in SLBBP, NSLBBP, and LVSP as the difference between the first and last activation (15). There is no doubt that Stim-LVAT of LVSP is significantly longer than that of LBBP, implying that the LV excitation in LVSP occurs later than in LBBP. As a result, the improved interventricular synchronization of LVSP is attributable to greater overlap of LV and RV activation produced by delayed activation of both the LV and the RV (18).

Curila et al. only evaluated the LBBP with unipolar pacing configuration, not bipolar pacing configuration (15). Lin et al. developed a bilateral bundle branch area pacing strategy that involves stimulating the cathode and anode in various pacing configurations to capture both LBB and right bundle branch (RBB) area, which can diminish delayed right ventricular activation caused by LBBP and result in more physiological ventricular activation (19). It is essentially LBBP with bipolar pacing configuration (Figure 1C), with the cathode tip capturing LBB and the anode ring capturing RBB area. Shimeno et al. also revealed that the terminal R′/r′ wave duration of LBBP with bipolar pacing configuration is shorter than that of LVSP, presumably due to the contribution of the anodal capture during bipolar pacing (17). In addition, some previous studies and case reports have shown that LBBP can shorten the QRS duration of intrinsic RBBB or even completely correct RBBB (19–23), while LVSP cannot, but the underlying mechanism remains unclear and needs further study.

## Conclusion

Compared with LVSP, LBBP is a more ideal pacing strategy for CRT, and many studies have confirmed its safety, stability and efficacy. Future study will focus on how to diminish RBBB associated with LBBP in order to obtain better physiological interventricular synchrony. For example, adjusting the atrioventricular delay to combined LV stimulation by LBBP with intrinsic RV excitation in patients with normal RBB conduction, or modifying the interelectrode distance of pacing lead to better complete bilateral bundle branch area pacing in patients with RBBB. Although LVSP in close proximity to LBB can be an alternative choice, clinically, this is essentially NSLBBP. The pacing output necessary to convert LVSP to NSLBBP, on the other hand, had not been investigated, and it was unknown if this output would have an adverse effect on pacemaker battery longevity. The long-term clinical effects of LVSP and LBBP remains unclear. Current studies solely examine the differences in electrophysiologic characteristics between LVSP and LBBP, such as Stim-LVAT, QRS duration, terminal R' wave duration, QRS area, etc. In the future, it will be necessary to evaluate the echocardiographic activation of LVSP and LBBP, encompassing not only intraventricular synchronization, but also interventricular synchronization.

## Author Contributions

KZ wrote the original manuscript and conceptualized the idea. DC and QL supervised and wrote and edited the manuscript for publication. All authors contributed to the article and approved the submitted version.

## Funding

This work was supported by Research Grant No. 3502Z20214ZD1165 from Xiamen Municipal Bureau of Science and Technology.

## Conflict of Interest

The authors declare that the research was conducted in the absence of any commercial or financial relationships that could be construed as a potential conflict of interest.

## Publisher's Note

All claims expressed in this article are solely those of the authors and do not necessarily represent those of their affiliated organizations, or those of the publisher, the editors and the reviewers. Any product that may be evaluated in this article, or claim that may be made by its manufacturer, is not guaranteed or endorsed by the publisher.
